# Systematic review and meta-analysis: Which pitfalls to avoid during this process

**DOI:** 10.1590/S1677-5538.IBJU.2020.0746

**Published:** 2020-12-20

**Authors:** Valeria Granados-Duque, Herney Andrés García-Perdomo

**Affiliations:** 1 Hospital Universitario Del Valle Evaristo Garcia Cali Colômbia Hospital Universitario Del Valle Evaristo Garcia, Cali, Colômbia; 2 Universidad Del Valle Cali Colômbia Universidad Del Valle, Cali, Colômbia

## INTRODUCTION

In our daily life, we face with decision-making and treatment choice, which, according to our ability to choose reliable sources and evidence-based literature allows us to select the best diagnostic and therapeutic alternative for our patients.

Publications in medical literature have increased over the years, and systematic reviews (SRs) have become progressively popular in medicine. Clinicians read them as an efficient manner of keeping up-to-date with their content area; they are a useful starting point for guideline development, helping to improve clinical practice (1, 2).

### 

#### What is the matter?

This starting point, part of the uncertainty, insomuch as change is more likely to occur if collective uncertainty exists; this doubt often reflects variations in medical practice. Asking a question that has already been answered by common sense or by powerful empirical evidence is of little use unless evidence suggests that the real answer is wrong. There are unknown data or a lack of usable data for some important questions, thereby demonstrating the demand for future research (1–3).

Systematic reviews and meta-analyses are essential tools for summarizing evidence accurately and faithfully. They might help clinicians to keep up-to-date; provide evidence for policymakers to evaluate risks, benefits, and harms of health maintenance behaviors and interventions; gather together and summarize related research for patients and their care-providers; give a starting point for clinical practice guideline development; and provide summaries of previous research for funders wishing to support new research (1–3).

Addressing a focused clinical question in a structured and reproducible manner, using systematic and explicit methods to identify, select, and critically appraise relevant research, and collecting and analyzing data from the studies that are included in the review, attempt to collate all empirical evidence that fits pre-specified eligibility criteria to answer a specific research question. It uses explicit, systematic methods that are selected to minimize bias, thus providing reliable findings from which conclusions can be drawn and decisions made. Otherwise, meta-analysis refers to the use of statistical techniques in a systematic review to integrate the results of included studies (3).

A mistake that is usually made in practice is to confuse systematic reviews with meta-analyzes, these two concepts have many important differences: Systematic Reviews attempts to collate all empirical evidence that conforms to pre-specified eligibility criteria to answer a particular research question. It uses explicit, systematic methods that are selected with a view to minimizing bias; this includes a comprehensive, exhaustive search for primary studies on a focused clinical question, selection of studies using clear and reproducible eligibility criteria, critical appraisal of primary studies for quality, and synthesis of results according to a predetermined and explicit method; thus providing reliable findings from which conclusions can be drawn and decisions made. In the other hand, Meta-analysis (MA) use statistical techniques to integrate and summarize the results of included studies. MA can provide more precise estimates of the effects of health care than those derived from the individual studies included within a review. It is a two-stage process. The first stage involves the calculation of a measure of the treatment effect, with its 95% confidence intervals (CI) for each study. The summary statistics that are usually used to measure treatment effect include the odds ratios (OR), relative risks (RR), risk differences (RD), Hazard Ratios (HR) and mean differences (MD). In the second stage of meta-analysis, authors calculate an overall treatment effect based on the weight of each study, and a model (random or fixed) according to the heterogeneity (3, 4).

#### Issues to point out

- Some of the mistakes usually made when conducting a study are:
- Reviews did not report key aspects of systematic review methodology, thus impairing confidence in their results and conclusions.

Even when the possibility of publication bias is assessed, there is no guarantee that systematic reviewers have assessed or interpreted it appropriately.

Unfortunately, there is considerable evidence that key information is often poorly reported in systematic reviews, thus diminishing their potential usefulness. In this sense, evidence-based practice (EBP) anticipates methodologies and processes to identify evidence of whether certain treatment or diagnosis is effective, strategies to assess the quality of studies, and mechanisms to implement it with caution, and to conduct according to scientific precepts (3–6).

Traditional literature reviews (nowadays called narrative reviews) have been criticized for a long time because the bibliographic search and study selection method is not standardized and explicit. The results obtained through such reviews are biased, do not exhaust all the literature available about the theme, do not have a critical appraisal of the literature, and are usually inconclusive (7, 8).

The Preferred Reporting Items for Systematic Reviews and Meta-Analyses (PRISMA) is an evidence-based minimum set of items for reporting in systematic reviews and meta-analyses. PRISMA focuses on the reporting of reviews evaluating randomized trials, but can also be used as a basis for reporting systematic reviews of other types of research, particularly evaluations of interventions. It helps authors to report a wide array of systematic reviews to assess the benefits and harms of a health care intervention. It provides further details regarding its background and development. This accompanying document explains the significance and rationale for each of the 27 checklist items (3, 4).

#### Step by step

Initially, researchers must determine what type of intervention want to carry out and the population to which it will be directed, one useful strategy is establishing the PICO question tool. It focuses on the Population, Intervention, Comparison and Outcomes. It helps readers as it provides key information about the focus of the review. Specifying the design(s) of the studies included, as shown in the examples, may also help some readers and the searching databases (9).

The research question must address what is important to patients and clinicians; it must contribute to the community, solving a question, giving a new point of view. There are systematic reviews which cannot include any relevant studies and are referred to as an “empty review”. They are important to scientific knowledge, but they do not usually help the clinician in the decision-making process. Perhaps, it had a highly specific PICO question and an overly stringent methodological inclusion criterion, which is important but with no relevant results for clinical settings (3, 4).

Secondly, write a protocol, register, and create a searching strategy. A protocol is an essential part of the review process and should include sufficient data to enable independent replication of the methods. Adherence to a pre-defined protocol is a key method with which to avoid the introduction of selection bias, as it ensures that all-important decisions have been made in advance of knowledge of the results. Even so, sometimes, it is usual to find changes to the protocol to improved quality, which is important to keep in mind since it is liable to fall into the bias (9).

A wide range of health-related bibliographic databases exists; three bibliographic databases are generally considered to be the most important sources for searching: Central, Medline and Embase (3, 9, 10). Employing more than one database is a useful tool, based on a search strategy and whose objective is to solve, support or justify the hypothesis. Searches should be motivated directly by the eligibility criteria for the review, and, significantly, all types of eligible studies are considered when planning the search (11).

Further, registration of a systematic review, typically with a protocol and registration number, is not yet common but may reduce the risk of multiple reviews addressing the same question, reduce publication bias, and provide greater transparency when updating systematic reviews (4, 12–14).

Thirdly, establish a selection and information collection criteria. The knowledge of the eligibility criteria is essential in appraising the validity, applicability, and comprehensiveness of a review is based on the search strategy; which is conducted according to the standards established with the PICO tool. In this manner, all these steps must be performed for at least two investigators to reduce the possibility of eliminating the relevant report. Indeed, the authors should describe these methods, including any steps taken to reduce bias and mistakes during data collection and data extraction. Also, there must be standardized protocols for data collection, including training of study personnel; minimizing inter-observer variability when multiple individuals are gathering and entering data (4, 14–17).

Fourthly, assess the risk of bias and quality of the included studies. Bias refers to systematic error, meaning that multiple replications of the same study would reach the wrong answer on average. It can occur at any phase of research, including study design, data collection, data analysis and publication. There are multiple instruments or validated scales to avoid this pitfall. The most common tools are Cochrane risk of bias tool, Newcastle - Ottawa Scale (NOS), MINORS, ROBINS-I, QUADAS2 and Grading of Recommendations Assessment, Development and Evaluation (GRADE). Authors must choose according to the kind of the included study (14, 16–20).

Fifthly, include all languages. Selection bias is an important pitfall in recent systematic reviews. Multiple publications only include English language; however, what Cochrane recommends is including all kinds of languages for lowering this kind of bias (21).

Finally, in order to critically appraise the SRs, there are useful strategies: The A Measurement Tool to Assess Systematic Reviews (AMSTAR) created in 2007, is an 11-item tool that has been developed to evaluate SR quality and determine whether the most important elements are reported. AMSTAR-2 an update to the original AMSTAR tool allows a more detailed evaluation of SRs that also includes non-randomized studies; the latter contains a questionnaire with 16 domains, through which the quality of the systematic reviews can be evaluated, including defects that may have arisen due to the misconduct of the other instrument of risk of bias in SRs. In this sense, it differs from another instrument for risk of bias in SR, the ROBIS, which is a triphasic instrument that focuses on the risk of bias introduced. ROBIS is an effective tool for assessing the risk of bias in systematic reviews, but compared to AMSTAR and AMSTAR 2 with the ROBIS tool; the last shows lower agreement and it is more difficult to use (17, 19, 20, 22–17).

There are also quicker ways to assess systematic reviews, as described by Taylor et al. (28). Ten questions to easily assess systematic reviews:

1. Is the study question relevant?
2. Does the study add anything new?
3. What type of research question is being asked?
4. Was the study design appropriate for the research question?
5. Did the study methods address the most important potential sources of bias?
6. Was the study performed according to the original protocol?
7. Does the study test a stated hypothesis?
8. Were the statistical analyses performed correctly?
9. Do the data justify the conclusions?
10. Are there any conflicts of interest?

In conclusion, systematic reviews and meta-analyses have become increasingly popular in medicine, and are essential tools for summarizing evidence accurately and reliably. They help clinicians keep up-to-date, and to create elements to evaluate information in an organized and structured way, allowing the results to be reproducible. Following the standard rules for performing systematic reviews will limit the possibility of making bias and will increase the transparency and reliability of the results. Hence, understanding research bias allows readers to critically and independently review the scientific literature and avoid treatments that are suboptimal or potentially harmful.
